# A novel splice variant of folate receptor 4 predominantly expressed in regulatory T cells

**DOI:** 10.1186/1471-2172-13-30

**Published:** 2012-06-13

**Authors:** Yi Tian, Guoqiang Wu, Jun-Chao Xing, Jun Tang, Yi Zhang, Ze-Min Huang, Zheng-Cai Jia, Ren Zhao, Zhi-Qiang Tian, Shu-Feng Wang, Xiao-Ling Chen, Li Wang, Yu-Zhang Wu, Bing Ni

**Affiliations:** 1 Institute of Immunology, PLA, Third Military Medical University, Chongqing 400038, Peoples Republic China; 2 Department of general surgery, the general hospital of ShenYang military area command, ShenYang 110016, Peoples Republic of China; 3 Department of Dermatology, the 105th Hospital of PLA, Hefei 230001, Peoples Republic of China

**Keywords:** Folate receptor 4, Variant, Regulatory T cells, Proliferation

## Abstract

**Background:**

Regulatory T cells (Tregs) are required for proper maintenance of immunological self-tolerance and immune homeostasis. Folate receptor 4 (FR4) is expressed at high levels in transforming growth factor-beta (TGF-β)-induced Tregs and natural Tregs. Moreover, antibody-mediated targeting of FR4 is sufficient to mediate Treg depletion.

**Results:**

In this study, we describe a novel FR4 transcript variant, FR4*D3*, in which exon 3 is deleted. The mRNA of FR4*D3* encodes a FR4 variant truncated by 189 bp. FR4*D3* was found to be predominantly expressed in CD4^+^CD25^+^ Treg cells. Overexpression of FR4*D3* in CD4^+^CD25^+^ Treg cells *in vitro* stimulated proliferation, which may modulate the ability of these cells to bind and incorporate folic acid.

**Conclusions:**

Our results suggested that high levels of FR4*D3* may be critical to support the substantial proliferative capacity of Treg cells.

## Background

The folate receptor (FR), also known as the folic acid (FA) binding protein, is responsible for binding of 5-methyltetrahydrofolate (5-MeTHF) with high affinities (~100 pM Kd), thereby playing an important role in the uptake of serum folates by cells expressing this receptor [1,2]. Four isoforms of the human FR, FR-α, -β, -γ and –δ, have been identified and characterized. FR-α is predominantly expressed on epithelial cells [3,4]. FR-β is expressed only on activated macrophages and the surfaces of malignant cells of hematopoietic origin [5]. FR-γ was identified as a secretory protein from hematopoietic tissues, and demonstrated to have a much lower affinity for FA than FR-α [6,7]. FR-δ has proven difficult to detect in human tissues, suggesting a highly restricted spatial/temporal expression pattern, presence of a pseudogene, or predominance of an alternatively spliced variant [8]. Recent data, however, has indicated that FR-δ may be expressed on regulatory T cells (Tregs) [9].

Analysis of amino acid sequences in the mouse led to the identification of homologues of human FR-α and FR-β, known as folic acid-binding protein 1 and 2 (Folbp1/FR1 and Folbp2/FR2) [10]. Utilizing a ‘genome database mining’ strategy, Spiegelstein *et al.* identified a third murine FR, Folbp3 (also called FR4), which is highly homologous to human FR-δ [8,11]. In addition to regulating FA uptake, FR4 has been hypothesized to play a potential role in immune responses based upon its expression profile, which includes spleen- and thymus-related lymphoid tissues and lymphocytes [8]. Indeed, FR4 has been demonstrated to be exclusively expressed in splenic lymphocytes, especially in the T lymphocytes, and in mature thymocytes; however, few studies have reported on the characterization of FR4 [8,11].

Tregs are required for proper maintenance of immunological self-tolerance and immune homeostasis [12]. Studies to identify molecular markers of Tregs determined that FR4 is expressed at particularly high amounts in natural (n)Tregs and plays an important role in the maintenance of the Treg phenotype [13]. In our previous study, we identified a FR4 cDNA splice variant (FR4v) with intron 3 (108 bp) retained from the FR4 gene and confirmed protein expression of this variant [14]. Here, we report the identification of another FR4 transcript variant, named FR4*D3*, with the full-length exon 3 (189 bp) deleted. The FR4*D3*-encoded protein was confirmed to be expressed on CD4^+^CD25^+^ Tregs by Western blotting and fluorescence-activated cell sorting (FACS) assays. Finally, the role of the FR4*D3* variant was investigated by overexpression in Treg cells *in vitro*.

## Methods

### Isolation of RNA, amplification and cloning of FR4*D3*

Six to eight-week-old female BALB/c mice were purchased from the Animal Center at the Chinese Academy of Medical Science(CAMS) branch in Beijing, China. All animals were maintained in pathogen-free conditions. All of the animal studies were approved by the Institutional Animal Care and Use Committee of the Third Military Medical University.

Total RNA was extracted from BALB/c mouse splenocytes using the TRIzol® reagent (Invitrogen, USA) according to the manufacturer's instructions. Approximately 1 μg of total RNA was reverse transcribed to cDNA using the High Fidelity PrimeScript™ reverse transcription-polymerase chain reaction (RT-PCR) kit (TaKaRa, Japan). The cDNA was then used as template to PCR amplify the FR4 coding DNA sequence (CDS) with the following primers corresponding to the ends of exons 2 and 5 of the mouse FR4 gene, respectively: mFR4-F, 5'-ATGGCACAGTGGTGGCAGAT-3'; mFR4-R, 5'-TCAGG GATGGAACAACAGGC-3'. The PCR reaction was carried out under the following thermal cycling conditions: 30 cycles of 98°C for 10 s and 68°;C for 40 s. The PCR amplicons were then subcloned into the pMD19-T vector (TaKaRa), and the clones were fully sequenced at Shanghai Sangon Biological Engineering Technology & Services Co., Ltd (China). The mRNA expression of FR4, FR4v and FR4*D3* genes in splenocytes, CD4^+^ T cells, and CD4^+^CD25^+^ T cells were detected using RT-PCR assays with the following primers: 5'-GGGACAAACTGCTCAGCGTCT-3' (forward) and 5'-AGACACCGCCCACTGTTCCT-3' (reverse).

To construct FR4- or FR4*D3-*expressing recombinant plasmids, the FR4 or FR4*D3* coding sequence was PCR-amplified from BALB/c mouse splenocytes RNA and cloned directly into the pCI-neo vector (Promega, USA). The following primers were used: Forward primer: 5'-CCGCTCGAGGCCACCATGGCACAGTGGTGGCAGAT-3'; Reverse primer: 5'-CGTGGGTGCTCTAGATCAGGGATGGAACAACAGGC-3'.

### FACS analysis

Approximately 1 × 10^8^ splenocytes were stained with anti-CD4-FITC, anti-CD25-PE and anti-CD8- PerCP-Cy5.5 antibodies (all from eBioscience, USA). The stained cells were separated by FACS into batches of CD4^+^CD25^+^ T cells, CD4^+^CD25^-^ T cells, and CD8^+^ T cells with a FACS-Aria high-speed cell sorter (BD Biosciences, USA). The isolated CD4^+^CD25^+^ T cells were stained with intracellular anti-FOXP3-APC antibody (eBioscience, USA) and then detected by the FACS-Aria high-speed cell sorter.

### Western blotting analysis

Isolated splenocytes and T cell subsets CD4^+^CD25^–^, CD4^+^CD25^+^, or CD8^+^ were lysed with the T-PER® Tissue Protein Extraction Reagent (Pierce, USA) at room temperature. After 5 min of lysis, the cell debris was removed *via* centrifugation at 12,000 rpm for 5 min at 4°;C and the lysates were treated with N-glycosidase before blotting [15]. The protein-containing supernatant was mixed with 4 × nonreducing lithium dodecyl sulfate (LDS) sample buffer (Invitrogen) and heated for 10 min at 70°;C. The protein samples (10–50 μg) were resolved by electrophoresis through NuPAGE® Novex 10% Bis-Tris mini-gels (Invitrogen) and transferred onto a polyvinylidene fluoride (PVDF) membrane (Millipore, Germany). After blocking of non-specific sites, the membranes were incubated with primary antibody (1:1,000 rat anti-mouse FR4; Abcam, United Kingdom) at 4°;C overnight, followed by incubation with secondary antibody (1:10,000 goat anti-rat IgG; Abcam) for 1 h. Immunoreactive bands were observed using the enhanced chemiluminescence (ECL) detection reagents (Amersham Biosciences, United Kingdom), according to the manufacturer’s instructions. β-actin was detected as an internal control (primary antibody: rabbit anti-mouse β-actin, secondary antibody: goat anti-rabbit IgG; both from Abcam).

The pCI-neo-FR4 or pCI-neo-FR4*D3* vector (0.5 μg) was added to 2 × 10^4^ FACS-sorted CD4^+^CD25^+^ Tregs resuspended in 100 μL of mouse T cell Nucleofector solution and electroporated using the 96-DN-100 program on the Nucleofector instrument (Lonza, Germany). After 24 h of culture in serum-free conditions, the expressions of FR4 and FR4*D3* proteins in the respective transfected cells were analyzed with Western blotting assay using anti-mouse FR4 (Abcam) as the primary antibody. The immunoreactive bands were scanned and densitometric analysis was performed by Bio-Rad Quantity One software.

### Proliferation assays

The FACS-sorted CD4^+^CD25^+^ Tregs were stimulated with various concentrations of FA (0, 2, 4, 6, 8, 10 ng/mL; Sigma-Aldrich, USA) in the presence of interleukin (IL)-2 (300 U/mL; R&D Systems, USA), anti-CD3 (5 μg/mL; BD Biosciences)/anti-CD28 (2 μg/mL; BD Biosciences) (pre-coated and soluble, respectively). Cultures were incubated for three days and labeled with 0.5 μCi (0.0185 MBq) [^3^H]thymidine ([^3^H]TdR; Hartmann Analytics, Germany) during the last 18 hours of incubation. [^3^H]TdR incorporation was measured by a liquid scintillation counter (Top Count; Perkin Elmer, Germany). All assays were performed in triplicate.

The CD4^+^CD25^+^ Tregs transfected with pCI-neo-FR4 or pCI-neo-FR4*D3* plasmids were seeded in 96-well U-bottom plates pre-coated with anti-CD3(5 μg/mL) and co-stimulated for three days with soluble anti-CD28 (2 μg/mL), with or without IL-2 (300 U/mL) or FA (4, ng/mL). During the last 18 hours of culture, cells were labeled with 0.5 μCi (0.0185 MBq) [^3^H]thymidine. [^3^H]thymidine incorporation was measured by a liquid scintillation counter. All assays were performed in triplicate.

### Statistical analysis

Relative comparisons of FR4 and FR4*D3* proteins’ intensity abundance between the transfected cells and untransfected cells detected by Western blot assay and the differences in proliferation of sorted CD4^+^CD25^+^ Tregs were analyzed by means of the 2-tailed Student’s *t*-test. *P-*values less than 0.05 were considered significant.

## Results

### Identification of a novel FR4 isoform

**Figure 1:**
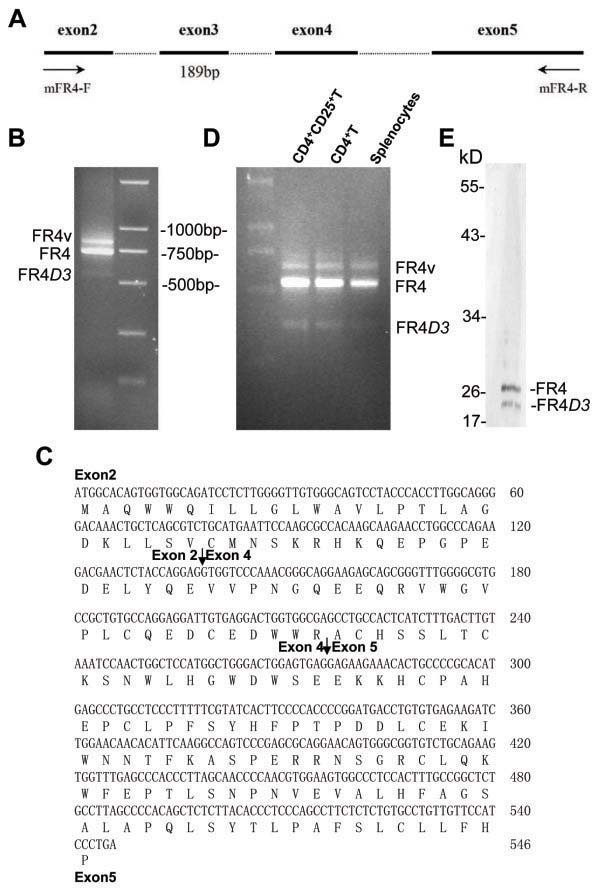
**Identification of a novel truncated FR4 variant from BALB/c mouse splenocytes.** (A) Schematic diagram of the mouse FR4 gene. Exons are indicated by solid lines, and introns by dashed lines. Positions of primers used for PCR amplification are shown by arrows. (B) RT-PCR detection of full-length CDS of FR4 and FR4*D3* genes in mouse splenocytes. (C) RT-PCR detection of FR4 and FR4*D3* mRNA in splenocytes, CD4^+^ T cells, and CD4^+^CD25^+^ T cells. (D) The nucleotide and predicted amino acid sequences of the novel mouse FR4*D3* CDS. The 189 bp exon 3 was deleted and did not cause frame shift mutation. The exons are indicated in bold and the exon-exon junction sites are indicated by arrows. (E) Western blotting assay showing FR4 and FR4*D3* proteins detected by rat anti-mouse FR4 monoclonal antibody (mAb). Here, 50 μg splenocyte lysates were treated with N-glycosidase before blotting [15].

When we amplified the full-length FR4 CDS from murine splenocytes by RT-PCR with the primer pair mFR4-F and mFR4-R (Figure 1A), three distinctive transcripts were amplified. The sizes of the two largest transcripts corresponded to the full-length FR4 (735 bp) and the variant FR4v (843 bp) [14]; however, the third transcript appeared to be a truncated form of FR4 (546 bp) (Figure 1B). The 546 bp PCR product was isolated, cloned and sequenced. Results showed that the 546 bp band’s sequence was almost identical to the FR4 CDS, except with 189 bp of contiguous sequence deleted. Alignment analysis with FR4 mRNA sequence revealed that the missing 189 bp sequence corresponded to the entire exon 3 of the reported FR4 mRNA (Figure 1C). This newly identified splice variant of the FR4 gene was designated as FR4*D3*. Furthermore, the results indicated that the mature mRNAs of FR4*D3*, FR4v, and FR4 were generated from the same pro-mRNA. We then investigated the mRNA expression of FR4 gene variants in splenocytes, CD4^+^ T cells, and CD4^+^CD25^+^ T cells, respectively. The RT-PCR assays revealed that all FR4 variants were expressed in these cell types, but the relative abundance of FR4*D*3 was lower than the other variants (Figure 1D).

Since the 189 bp-length exon 3 is composed of exactly 63 triplet codes of the FR4 mRNA, its elimination will not change the correct translation of FR4*D3* mRNA. Therefore, Western blotting assay was used to determine whether the FR4*D3* protein was expressed in mouse spleen cells. As shown in Figure 1E, the anti-FR4 antibody detected both the full-length FR4 and the truncated FR4*D*3 variant in mouse splenocytes, whose predicted molecular masses were approximately 28 kDa and 21 kDa, respectively. The sequence and theoretical translation of this novel cDNA were submitted to GenBank and can be found under accession numbers EU326439.1 and ABY56299.1, respectively.

### Predominant expression of FR4*D3* in CD4^+^CD25^+^ regulatory T cells

**Figure 2:**
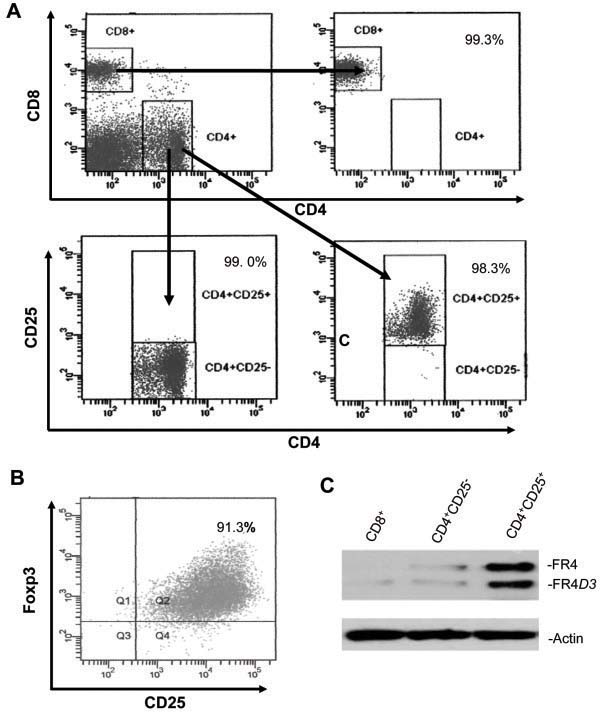
**Expression of the novel FR4*D3 *variant in splenic T cell sub populations.** (A) Isolation and purity (%) of splenic T cell subpopulations. (B) Purity of Foxp3-positive cells in isolated CD4^+^CD25^+^ T cells. (C) FR4*D3* protein expression in particular T cell subsets was detected by Western blotting assay using anti-mouse FR4 mAb . Here, 10 μg of total cell extracts was treated with N-glycosidase before blotting. β-actin protein was detected as an internal control.

It has been reported that natural Treg cells constitutively express higher amounts of FR4 [13]. In order to examine whether the FR4*D3* exhibits a differential distribution in T cell subpopulations, high purity populations of specific T cell subtypes were obtained by FACS sorting. The purity of isolated CD8^+^, CD4^+^CD25^–^, and CD4^+^CD25^+^ T cells reached 99.3%, 99.0%, and 98.3%, respectively (Figure 2A). After sorting, we detected the purity of Foxp3-positive cells in the isolated CD4^+^CD25^+^ T cells and found that the Foxp3-positive cells reached ~91%, suggesting that most of the isolated CD4^+^CD25^+^ T cells were Tregs (Figure 2B). Western blotting analysis of these subtypes showed that FR4 and FR4*D3* proteins were expressed in CD4^+^CD25^+^ Tregs at a higher level than in either CD4^+^CD25^-^ or CD8^+^ T cells (Figure 2C). These results suggested that FR4*D3* may play a redundant or unique functional role in CD4^+^CD25^+^ Tregs.

### Effect of overexpression of FR4*D3* on proliferation of CD4^+^CD25^+^ T cells

**Figure 3:**
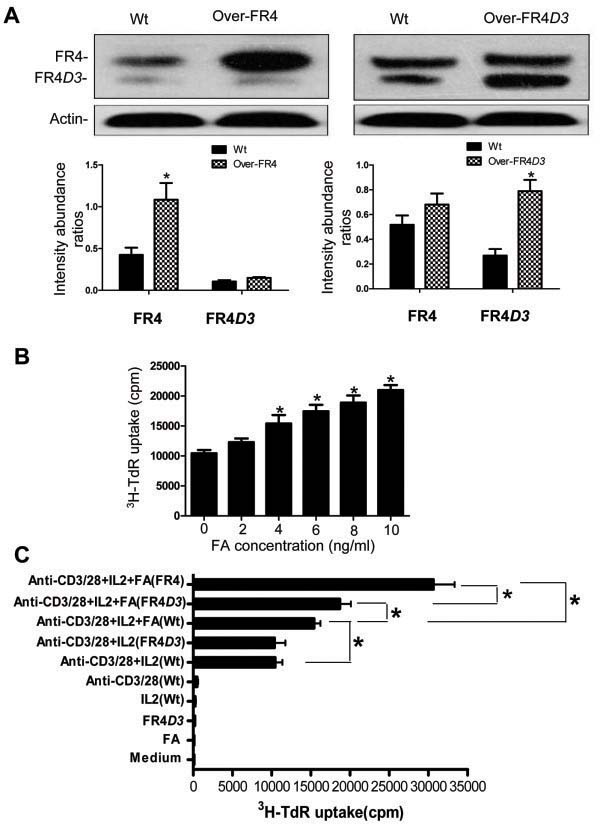
**Proliferation of sorted CD4**^**+**^**CD25**^**+**^**Tregs.** (A) Overexpression of FR4 or FR4*D3* protein in sorted CD4^+^CD25^+^ Tregs. Wt, wild type cells; Over-FR4, overexpression of FR4; Over-FR4*D3*, overexpression of FR4*D3*. Each column represents the intensity abundance ratio of the FR4 or FR4*D3* band *versus* the actin band in each lane. Panels show a representative of three independent experiments. Error bars represent the standard deviation from three independent experiments. *, *P* < 0.05, vs. wild type cells. (B) FA dose-response of Tregs’ proliferation. The CD4^+^CD25^+^ Tregs were stimulated with various concentrations of FA (0, 2, 4, 6, 8, 10 ng/mL) in the presence of IL-2, anti-CD3/anti-CD28 (pre-coated and soluble, respectively). Panels show a representative of three independent experiments. Error bars represent the standard deviation from triplicate wells.*, *P* < 0.05 vs. untreated cells. (C) The FR4 or FR4*D3* CDS was cloned directly into the pCI-neo vector and transiently transfected into FACS-sorted CD4^+^CD25^+^ Tregs, followed by stimulation with or without combinations of FA, IL-2, and anti-CD3/anti-CD28 (pre-coated and soluble, respectively). Wt represents no overexpression of either FR4 or FR4*D3*; FR4 represents the overexpression of FR4; FR4*D3* represents the overexpression of FR4*D3*. Panels show a representative of three independent experiments. Error bars represent the standard deviation from triplicate wells. *, *P* < 0.05.

The capacity to efficiently bind and incorporate folic acid is linked to cellular proliferation, and indeed overexpression of folate receptors is a characteristic feature of certain tumors [16]. It has been reported that FR4 is not merely a marker of natural Treg cells but is also functionally essential for their maintenance since blockade of FR4 was sufficient to reduce natural Treg cells *in vivo*[13]. In order to investigate the function of FR4 and FR4*D3*, we respectively overexpressed each in the isolated CD4^+^CD25^+^ Tregs. Results showed that the level of FR4 or FR4*D3* protein was much higher in transfected cells than in untransfected cells (Figure 3A). We then examined the proliferative ability of FACS-sorted CD4^+^CD25^+^ Tregs in response to stimulation with FA, in the presence of IL-2, anti-CD3/anti-CD28 (pre-coated and soluble, respectively) *in vitro*. Results should that only simultaneous stimulation with anti-CD3/anti-CD28 in the presence of IL-2 could abrogate the anergic state of CD4^+^CD25^+^ Tregs. Moreover, the proliferative ability of these cells was further enhanced by addition of FA to the stimulation mixture in a dose-response manner (Figure 3B), and FA mediated more proliferation than simply overexpressing the FR4*D3* gene (Figure 3C). However, the proliferation capacity of cells induced with FA + IL-2 + anti-CD3/anti-CD28 was even further enhanced by overexpression of FR4 or FR4*D3* (Figure 3C), indicating that both receptors have a substantial capacity to uptake the FA substrate. The wild-type (Wt) FR4 appeared to have stronger ability to uptake FA than the FR3*D3* variant since overexpression of the former showed higher proliferative capacity in the presence of the FA substrate (Figure 3C).

## Discussion

In this study, we have identified a novel alternative splicing variant of the FR4 gene, named FR4*D3*, which lacks the entire exon 3 of the FR4 gene. FR4*D3* proteins were found to be expressed in CD4^+^CD25^+^ Tregs at a higher level than in CD4^+^CD25^-^ or CD8^+^ T cells. Furthermore, overexpression of FR4*D3* in FACS-sorted CD4^+^CD25^+^ Tregs was found to enhance proliferative capacity of these cells *in vitro*.

Recent studies have demonstrated that nearly every multi-exon gene, including “constitutively” spliced genes [17], produces alternative mRNA isoforms [18]. Although many of these isoforms have important functional roles, it is clear that some of these mRNAs are produced by errors that occur during the splicing process [19]. In fact, different RNA quality controls have evolved to recognize and degrade such errors. For those mRNAs that escape detection and destruction, expression may be at such low levels that the new mRNA isoforms may be tolerated by the cell, eventually representing an evolutionary precursor [20,21].

Previous studies have suggested that FR4 is exclusively expressed by splenic lymphocytes, especially the T lymphocytes, and mature thymocytes. Subsequent study identified particularly high expression in the nTregs T cell subpopulation [13]. In our current study, we found that FR4*D3* was expressed in CD4^+^CD25^+^ Tregs at a higher level than in either CD4^+^CD25^-^ or CD8^+^ T cells, suggesting that the expression of FR4*D3* was cell type-specific. Retroviral transduction of Foxp3, which can functionally and phenotypically convert normal T cells to natural Treg-like cells [22,23], has revealed that the FR4 expression was proportional to that of Foxp3 in Foxp3-transduced CD25^-^CD4^+^ T cells, suggesting that Foxp3 can control, either directly or indirectly, the expression of FR4 in natural Treg cells [13]. Further research is necessary to determine whether transduction with Foxp3 is also sufficient to mediate FR4*D3* up-regulation.

As previously described for CD4^+^CD25^+^ Tregs, the anergic state of a pure CD4^+^CD25^+^ Treg subpopulation is abrogated after simultaneous stimulation of T cell receptors (TCRs) and CD28 in the presence of IL-2; neither IL-2 nor plate-bound anti-CD3 combined with soluble anti-CD28 could resolve the cells’ unresponsiveness [24,25]. Furthermore, the capacity to efficiently bind and incorporate folic acid is linked to cellular proliferation, in both normal and tumorigenic conditions. In our current study, we found that overexpressing FR4*D3* or FR4 in CD4^+^CD25^+^ Tregs could further enhance the proliferation induced by FA, IL-2, and anti-CD3/anti-CD28 (pre-coated and soluble, respectively) *in vitro*. These results suggested that high expression of FR4*D3* could facilitate binding of FA and cellular uptake, which may be critical to support the substantial proliferative capacity of Tregs *in vivo*. Of course, the FR4*D3* may have other yet unrecognized functions, which will require further investigation. We also found that the FA + IL-2+ anti-CD3/anti-CD28 induced proliferative capacity of CD4^+^CD25^+^ Tregs overexpressing FR4*D3* was weaker than in CD4^+^CD25^+^ Tregs overexpressing FR4. These results suggested that the exon 3-related sequences contribute to optimal FA binding and/or cellular uptake mediated by FR4.

## Conclusions

In conclusion, we have identified a novel exon 3-deleted FR4 transcript variant, which encodes a 189 bp truncated protein that is predominantly expressed in CD4^+^CD25^+^ Treg cells. The high expression of FR4*D3* in CD4^+^CD25^+^ Tregs may modulate the ability of these cells to bind and incorporate folic acid, possibly in normal or pathogenic conditions that would benefit from enhanced proliferation of these cells.

## Competing interests

The authors have declared that no competing interests exist.

## Authors’ contributions

Yi Tian, Guoqiang Wu and Jun-Chao Xing carried out the isolation of RNA, amplification and cloning of FR4D3. Jun Tang and Yi Zhang carried out the FACS assay. Ze-Min Huang and Zheng-Cai Jia carried out the Western blotting analysis. Ren Zhao, Zhi-Qiang Tian and Li Wang carried out the proliferation assays. Xiao-Ling Chen and Shu-Feng Wang carried out the Statistical analysis. Yuzhang Wu and Bing Ni conceived the study, and participated in its design and coordination and drafted the manuscript equally. All authors read and approved the final manuscript.
